# Chemotherapeutic Potential of *Epimedium brevicornum* Extract: The cGMP-Specific PDE5 Inhibitor as Anti-Infertility Agent Following Long-Term Administration of Tramadol in Male Rats

**DOI:** 10.3390/antibiotics9060318

**Published:** 2020-06-11

**Authors:** Ahmed S. Abdelaziz, Mohamed A. Kamel, Amany I. Ahmed, Shimaa I. Shalaby, Salama M. El-darier, Amany Magdy Beshbishy, Gaber El-Saber Batiha, Suliman Y. Alomar, Dina M. Khodeer

**Affiliations:** 1 Pharmacology Department, Faculty of Veterinary Medicine, Zagazig University, Zagazig 44519, Egypt; makamel@zu.edu.eg; 2 Biochemistry Department, Faculty of Veterinary Medicine, Zagazig University, Zagazig 44519, Egypt; aialsayed@zu.edu.eg; 3 Physiology Department, Faculty of Veterinary Medicine, Zagazig University, Zagazig 44519, Egypt; Siabdallah@zu.edu.eg; 4 Botany and Microbiology Department, Faculty of Science, Alexandria University, Alexandria 21568, Egypt; salama.eldarir@alexu.edu.eg; 5 National Research Center for Protozoan Diseases, Obihiro University of Agriculture and Veterinary Medicine, Nishi 2-13, Inada-cho, Obihiro 080-8555, Hokkaido, Japan; amanimagdi2008@gmail.com; 6 Department of Pharmacology and Therapeutics, Faculty of Veterinary Medicine, Damanhour University, Damanhour 22511, Al Beheira, Egypt; 7 Doping Research Chair, Department of Zoology, College of Science, King Saud University, Riyadh 11495, Saudi Arabia; 8 Department of Pharmacology and Toxicology, Faculty of Pharmacy, Suez Canal University, Ismailia 41522, Egypt; dina_khoudaer@pharm.suez.edu.eg

**Keywords:** *Epimedium brevicornum* Maxim, tramadol, male fertility, andrological parameters, nitric oxide

## Abstract

*Epimedium brevicornum* Maxim (EbM) is a well-known Chinese herb that has been widely used for the treatment of several diseases. The main purpose of this study is to examine the role of *Epimedium brevicornum* extract in certain andrological parameters in rats as a natural modulator for adverse viewpoints associated with chronic administration of tramadol (TAM). Fifty rats were categorized into five groups. Untreated rats were known as Group I, whereas rats in Groups II and III were administered 2.43 g/kg/day of *E. brevicornum* extract and 50 mg/kg/day of TAM for 130 consecutive days, respectively. Both of Groups IV and V were administered TAM for 65 successive days, followed by concomitant use of both drugs for another 65 days, with the *E. brevicornum* extract at doses of 0.81 and 2.43 g/kg/day, respectively. TAM showed an injurious effect on sperm attributes, serum hormones, tissue malondialdehyde, superoxide dismutase, and nitric oxide. Elevation of the apoptotic marker Bax and a reduction of Bcl2 were recorded. Histopathological abnormalities have been reported in rat testicles. Rats treated with *E. brevicornum* extract with TAM showed an improvement in all the parameters tested. It could be presumed that *E. brevicornum* extract plus TAM exhibits a promising effect on the enhancement of male anti-infertility effects.

## 1. Introduction

**Figure 1:**
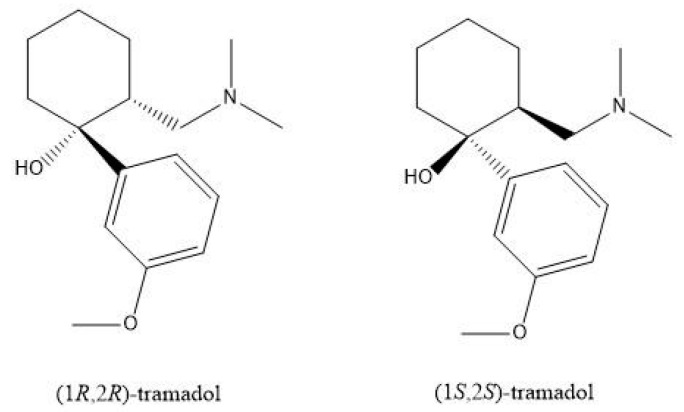
Chemical structure of tramadol hydrochloride.

Tramadol hydrochloride (TAM; Figure 1) is classified as a centralized pain reliever agent, used mainly to treat moderate and severe pain [1]. Tramadol was also found to be obtained through opioid and nonopioid mechanisms through sedative/analgesic action [2]. Efficient TAM is a racemic mixture of two enantiomers containing two well-defined and harmonious mechanisms: the (+) TAM is a µ-opioid selective agonist that inhibits serotonin reuptake to a higher degree and stimulates serotonin efflux in the brain, while the (‒) TAM enantiomorph inhibits the reuptake of noradrenaline [3]. Considering the fact of being opiate-related, TAM carries all possible risks from other opiates and shows a wide range of side effects, including dizziness, headache, drowsiness, vomiting attempts, bowel disease, sweating, pruritis, and CNS stimulation. These side effects are similar to opioids due to the affinity of tramadol to the micro-opioid receptor [4].

TAM has been reported to cause respiratory depression and psychological and physiological addiction [4]. Moreover, long-term administration of TAM results in an increase in oxidative stress that causes male infertility due to low levels of testosterone and neurodegenerative diseases such as Alzheimer’s disease [5,6]. There is a disturbing increase in the abuse of TAM, mainly among young people assuming that it improves sexual excitement and climax without the development of sexual dysfunctions [7], and small quantities for additional investigations and examinations have been implanted [8]. There is an urgent need for safer natural antioxidants to minimize the effects of oxidative and peroxidative damage of TAM on male infertility. Such natural antioxidants can prevent the issues associated with synthetic drugs, such as various side effects, treatment disappointment, and drug resistance.

**Figure 2:**
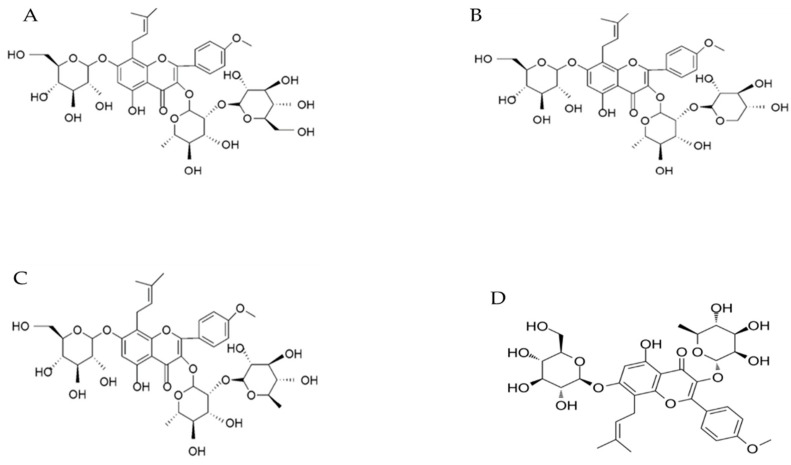
Chemical structures of the main bioactive components extracted from *Epimedium brevicornum*. (**A**) epimedin A, (**B**) epimedin B, (**C**) epimedin C, and (**D**) icariin.

*Epimedium* is a plant category of the Berberidaceae family, containing 63 plant species. *Epimedium brevicornum* Maxim (EbM) is a well-known Chinese herb that is related to the *Epimedium* genus and has been widely used for treating several ailments (e.g., osteoporosis, infertility, impotence, amnesia, senile functional disorders, and cardiovascular diseases) [9]. Chen and Chiu [10] claimed that intracavernous uptake of EbM extract might lead to a penile erection in rats. Wong et al. [11] defined the main bioactive components that were extracted from EbM, for instance, epimedin A, B, and C, and icariin (ICA; Figure 2A–D). ICA is the main flavonoid glycoside obtained from the aeronautical piece of the plant [12]. Furthermore, ICA shows a cGMP-specific PDE5 inhibitor capable of producing an orally efficient erectile dysfunction treatment agent [12,13]. Qureshi et al. [14] found that the administration of 300 to 500 mg/kg/day of *E. brevicornum* extract is a highly effective therapy for the treatment of impotency and reduces stress and depression. Furthermore, it was shown to be an excellent anxiolytic medicine and adaptogen for hormonal disorders. Within the current study, we assume that *E. brevicornum* extract might trigger an anti-infertility agent in rats. We tested the ameliorative effect of two different doses of *E. brevicornum* under the long-term administration of TAM.

## 2. Materials and Methods

### 2.1. Chemicals

*Epimedium brevicornum* extract as a source of icariin was purchased from Naturalin (code: NAT-091). The Molecular Structure and HPLC analysis chart is available on the company webpage (https://www.egypt-business.com/product/details/1617-epimedium-extract/35138). Tramadol hydrochloride 225 mg tablets were kindly given by the Laboratory of Forensic Sciences, Ministry of Justice (Cairo, Egypt). Its International Union of Pure and Applied Chemistry (IUPAC) name is as follows: (±) cis-2-{(dimethylamino) methyl}-1-(3-methoxyphenyl) cyclohexan-1-ol hydrochloride. All other chemicals used in this study were graded analytically.

### 2.2. Animal Use

Fifty adult male Wister rats of 200 ± 10 gm aged 4 to 5 months were collected from the Animal Research Unit, Faculty of Veterinary Medicine, Zagazig University, Egypt. The animals were housed in an environment free of pathogens, with controlled humidity, temperature (22 °C), and in an alternate 12 h light and dark cycle [15]. Two weeks before the experiment was conducted, the animals were allowed to acclimatize to the test facility conditions. The methodology was applied to all creatures dealt with, tested, and authorized by Zagazig University Research Center Institutional Animal Care and Use Committee (IACUC) under number ZU-IACUC/2/F/32/2018.

### 2.3. Model Building

Rats were caged into five groups, each of which consisted of ten rats. Group 1 was the control group that received an oral administration of 0.2 mL saline as the vehicle used for 130 consecutive days. Groups 2 and 3 were administered 2.43 g/kg/day of *E. brevicornum* and 50 mg/kg/day of tramadol for 130 consecutive days, respectively [5,16]. Groups 4 and 5 were received tramadol at 50 mg/kg/day for 65 days, followed by the herb extract at doses of 0.81 g/kg/day and 2.43 g/kg/day for another 65 days, respectively.

### 2.4. Sample Collection

At the end of the animal study, all rats were euthanized using an anesthesia system involving ether for serum and tissue collection [17]. In a BD Vacutainer PST II tube [18], blood samples were gathered, left for coagulation at room temperature, and centrifuged at 1200× *g* for 15 min to obtain serum. The serum samples were preserved at −20 °C to be used for various biochemical experiments. Each rat pair of testis and epididymis was quickly cut and rinsed with ice-cold saline, while the testicular tissue was dissected and frozen in liquid nitrogen for further examination. For the antioxidant measurement, a piece of one testis from each rat was homogenized using a WiseTis HG-15D homogenizer (Daihan Scientific Co., Seoul, Korea), while the other part of testis was maintained in 10% neutral buffered formalin to be used for histopathological studies.

### 2.5. Semen Assessment and Sperm Parameters

The caudal portion of the epididymis was precisely isolated from one testis and placed in a sterilized Petri dish containing 2 mL normal saline where it was macerated; the epididymal contents obtained were correctly treated as the semen. All the equipment and solutions used were prewarmed to 37 °C before used. A drop of the obtained suspension was then positioned on a clean glass slide and examined by under a light microscope at a high resolution of ×400 to assess the individual spermatozoa motility, whereas the total motile spermatozoa percentage was examined by various microscopical fields. Eosin-nigrosine-stained epididymal suspensions were prepared for the sperm viability assay to assess the proportion of live/dead sperm. An improved Neubauer hemocytometer tallying chamber was used to count the spermatozoa in the semen solution diluted with ordinary saline at a proportion of 1:4 and a few drops of formalin (40%) [19]. The sperm cell count exceeded the all-out spermatozoa number in four squares × 2500 × dilution factor. Eosin–nigrosine-recolored smears were analyzed under the oil immersion lens to evaluate sperm variation levels from the standard after an arbitrary analysis of 100 spermatozoa [20].

### 2.6. Hormonal Analysis

Enzyme-linked immunosorbent assay (ELISA) kits obtained from MyBioSource (San Diego, CA, USA) were used to examine the plasma luteinizing hormone (LH), testosterone (Tes), estradiol (E2), and follicle-stimulating hormone (FSH) levels. The absorbance was measured using an ELISA plate reader (DNM–9602; Beijing Perlong Medical Instrument Ltd., Beijing, China), as instructed by the manufacturer.

### 2.7. Assessment of NO and Antioxidant Scavenging Capacity

A suitable homogenate aliquot was ultracentrifuged for 30 min at 10,000× *g* at 4 °C and the supernatant obtained after centrifugation was used for the estimation of NO spectrophotometrically: catalase (CAT; EC 1.11.1.6), superoxide dismutase (SOD; EC1.15.1.1), and malondialdehyde (MDA; the lipid peroxidation marker), using Griess reagents, as described by Green et al. [21]. Spectrophotometrical determination of the MDA levels [22], CAT, and SOD activity at 570 and 480 nm wavelengths, respectively [23,24], was calculated using a Shimadzu-type spectrophotometer (UV 120-02). All antioxidant enzyme kits were carried out using Biodiagnostic tools (Biodiagnostic Company, Dokki, Giza, Egypt), following the fabricator’s stipulations.

### 2.8. Relative Quantitative RT-PCR Analysis

A detailed description of the protocols used has already been reported [25]. Real-time PCR (RT-PCR) was carried out according to the manufacturer’s instructions using an Mx3005P Real-Time PCR System (Agilent Stratagene, CA, USA) and 5x HOT FIRE Pol EvaGreen qPCR Mix Plus (Solis BioDyne, Tartu, Estonia). The primers used are listed in Table 1. The relative expression of each gene normalized to the housekeeping GAPDH was reported as fold change by 2^−ΔΔCT^, relative to control [26].

### 2.9. The Testes Morphology

The testicles were resolved in formalin (10%) and immersed in paraffin. Afterwards, 5-μm thick sections were prepared, stained with hematoxylin–eosin, and tested under an Olympus/3H light microscope (Olympus, Tokyo, Japan) [25].

### 2.10. Statistical Analysis

Statistical analysis was carried out using one-way ANOVA accompanied by a posthoc dunken test, SPSS 16 (SPSS, Chicago, IL, USA). Data were interpreted as mean ± SEM, and a *p*-value <0.05 was considered statistically significant.

## 3. Results

### 3.1. Efficacy of Oral Administration of E. brevicornum extract and/or Tramadol on Semen Parameters

**Figure 3:**
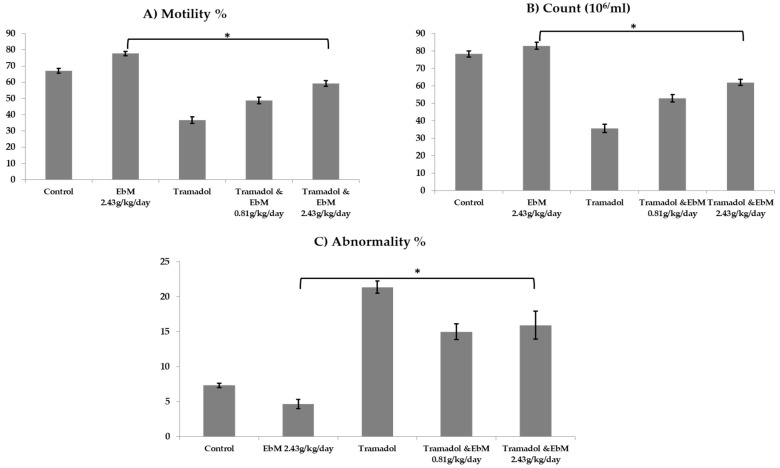
The efficacy of oral uptake of *E. brevicornum* extract and/or tramadol on semen parameters. Tramadol-administered rats resulted in a remarkable decrease in sperm motility (**A**) and count (**B**) and an improvement in sperm abnormalities (**C**) relative to the control group (* *p* ≤ 0.05). The effect of oral administration of *E. brevicornum* extract exerted a remarkable enhancement in sperm count and sperm motility, while the sperm abnormality associated with tramadol-treated rats was decreased (* *p* ≤ 0.05). Results are assessed using one-way ANOVA accompanied by Bonferroni’s test.

In order to detect the harm caused by the male conceptual framework, the sperm parameters were evaluated in the present investigation (Figure 3A–C). Concerning sperm parameters, sperm motility and count were remarkably increased, and abnormalities were reduced in the *E. brevicornum* group related to the control one, whereas substantial reduction in sperm motility and count and an improvement in abnormalities were observed in TAM-treated mice with regard to the control one (*p* < 0.05). Regarding the two *E. brevicornum*-administered groups, a remarkable increase was reported in the sperm count by 43.7% and 71.8% and motility by 66% and 98%, while reduction by 40% in the sperm abnormality was detected when compared with the TAM-treated rats.

### 3.2. Effect of E. brevicornum Extract and/or Tramadol Oral Administration on the Hormonal Assay

**Figure 4:**
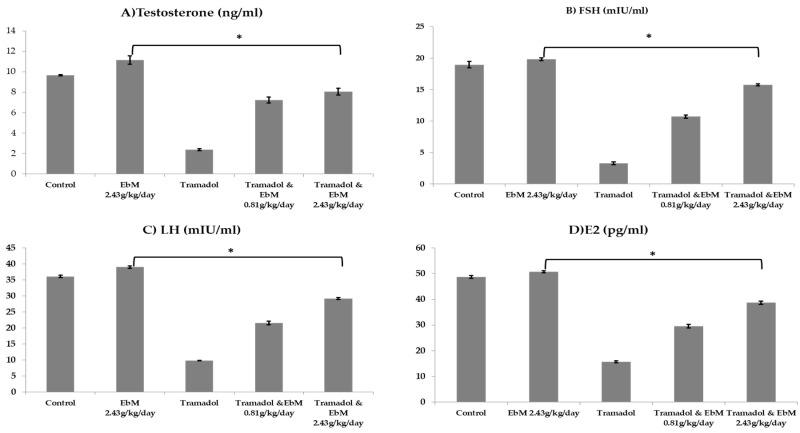
The effect of oral uptake of *E. brevicornum* extract and/or tramadol on hormonal assay. Results are presented as mean ± SEM and evaluated using one-way ANOVA accompanied by Bonferroni’s test for multiple comparisons. * indicates significant different at *p* < 0.05 from the tramadol-treated group. (**A**) Effect on testosterone level, (**B**) effect on FSH level, (**C**) effect on LH level, (**D**) effect on E2 level.

As appears in Figure 4A–D, *E. brevicornum* extract exhibited a significant impact on the sexual hormones of male rats in comparison with controls. In the interim, substantial decreases in Tes, FSH, LH, and E2 hormones were observed in the TAM group. In contrast, the herb extract-treated group showed exceptionally large increases in Tes, FSH, LH, and E2 hormones. The most ameliorative effects were observed in Group 5.

### 3.3. Effect of E. brevicornum Extract and/or Tramadol Oral Administration on NO and Antioxidant Status

**Figure 5:**
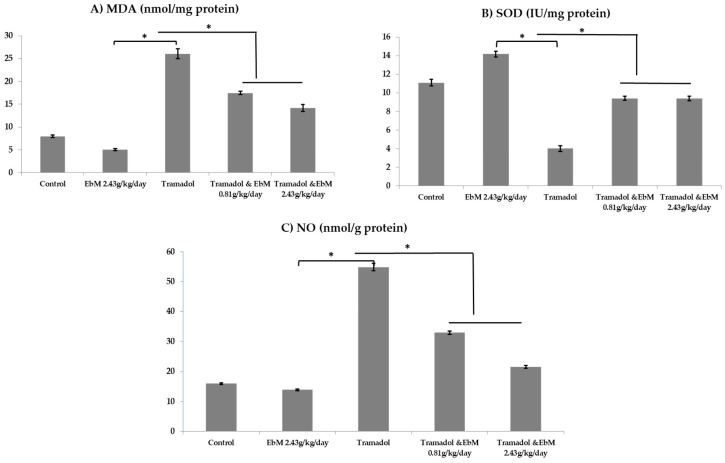
The effect of oral uptake of *E. brevicornum* extract and/or tramadol on malondialdehyde (MDA) (**A**), superoxide dismutase (SOD) (**B**), and nitric oxide (NO) (**C**) activity concentrations in testes of male rats. Results are represented as mean ± SEM and analyzed using one-way ANOVA accompanied by Bonferroni’s test for multiple comparisons. * Significant variation in the tramadol-treated group.

The conceptual framework power of males by TAM administration continues due to oxidative stress conditions in testicular tissues. The redox system activity has been assessed in the testes along these lines. In the TAM-treated rats, abnormal changes in oxidant and antioxidant enzyme activities and a decrease in the antioxidant activity were observed in the reduced SOD activities (*p* < 0.05) and increased MDA and nitric oxide (NO) production (*p* < 0.05), respectively. The two groups subjected to *E. brevicornum* were significantly reduced in all irregularities of oxidative stress (*p* < 0.05) with regard to TAM-treated rats, as shown in Figure 5A–C.

### 3.4. Expression of Apoptosis-Related Genes in Testicular Tissue

**Figure 6:**
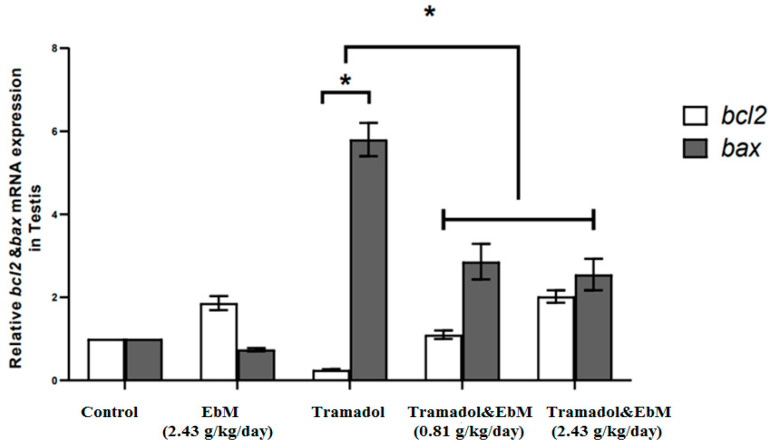
The effect of oral *E. brevicornum* extract and/or tramadol administration on the expression of apoptotic factor Bax and Bcl2 gene in testes of male rats. Tramadol-administered rats showed a substantial increase in apoptotic index (Bax) levels (by five-fold) relative to the control group (* *p* ≤ 0.05). The effect of oral administration of *E. brevicornum* extract exhibited a marked decrease in gene expression when compared to tramadol-treated rats (* *p* ≤ 0.05). Tramadol at a dose of 50 mg/kg/day caused a significant reduction in expression of Bcl2 (by 75%) when related to the control animals, followed by a substantial increase in *E. brevicornum* extract-treated groups. Results are presented as mean ± SEM and evaluated using one-way ANOVA accompanied by Bonferroni’s test.

An apoptotic index (Bax) demonstrated a five-fold increase in the TAM-treated group than that in the saline control one. At the same time, a considerable reduction in the gene expression to half was observed after *E. brevicornum* administration when compared to the TAM group (Figure 6). Tramadol at a dose of 50 mg/kg/day caused an exceptionally huge decrease in the expression of Bcl2 (by 75%) when related to the saline control group, followed by a remarkable rise in groups submitted to treatment with *E. brevicornum* alongside TAM.

### 3.5. Effect of Oral E. brevicornum Extract and/or Tramadol Administration on Testicular Histopathology

**Figure 7:**
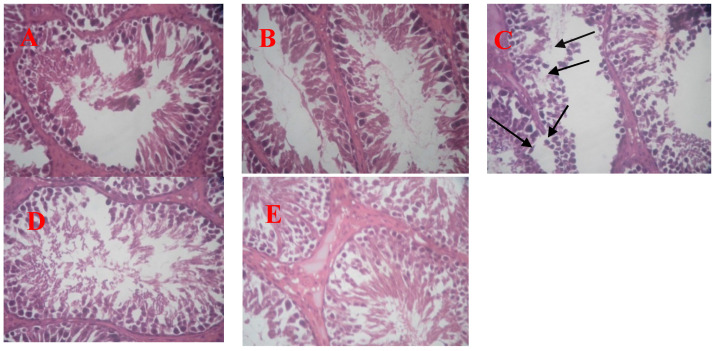
Plate (**A**): photomicrograph of the rat testis in the control rats shows normal, intact seminiferous tubules with normal lining epithelium and stratified seminiferous epithelium with normal interstitial connective tissue. Plate (**B**): photomicrograph of the rat testis in Group 2, which received *E. brevicornum* extract at a dose of 2.43 g/kg/day, show normal testicular parenchyma. Plate (**C**): photomicrographs of the testis in Group 3, which received tramadol at doses of 50 mg/kg/day and oral daily plate C, show necrosis, degeneration of the seminiferous tubule, sloughing of the stratified seminiferous epithelium, spermatogenic cells, and Sertoli cells, indicated with black arrows. Plate (**D**): photomicrographs of the rat testis of Group 4, which received tramadol at 50 mg/kg/day for 65 days, followed by TAM and *E. brevicornum* extract at a dose of 0.81 g/kg/day for another 65 days, show slight destruction and sloughing of spermatogenic cells, with slight hyperplasia of Leydig cells and slightly hyaline degeneration of the interstitial CT. Plate (**E**): photomicrographs of the testis in Group 5, which received tramadol at 50 mg/kg/day for 65 days, followed by tramadol and *E. brevicornum* extract at a dose of 2.43 g/kg/day for another 65 days.

Histopathological evaluation was carried out in all experimental groups, and the results are shown in Figure 7A–E. A typical histological structure of the seminiferous tubules in testis tissue was observed under light photomicrography, in sections prepared from control and *E. brevicornum*-treated groups (2.43 g/kg/day for 130 days). Necrosis and degeneration of the seminiferous tubule, (sloughing of the stratified seminiferous epithelium), spermatogenic cells and Sertoli cells, necrosis of some spermatogenic cells (especially spermatogonia, spermatocytes), and spermatids with a pyknotic nucleus appeared in animals receiving TAM 50 mg/kg/day for 130 consecutive days. Reasonable normalization of the testicular tissue, with a sustained normal histological structure of most of the seminiferous tubules, a moderate number of spermatozoa, and mild edema in the intertubular space, were observed in the tubular lumen of rats receiving both TAM and *E. brevicornum* in different doses, with a noticeable improvement in the higher proportion of *E. brevicornum*.

## 4. Discussion

Tramadol (TAM) is an atypical, midway-acting manufactured pain relief used to get rid of moderate to severe agony, with antinociceptive impacts that are interceded by a mixture of mu-opioid agonist impacts and inhibition of serotonin and norepinephrine reuptake [2]. In addition to cytotoxic drugs, different drugs can affect human fertility through various components. By modifying the hormones or nonhormonal systems of the hypothalamic–pituitary–gonadal axis, medicines can legitimately lead to sexual interruption and impairment of spermatogenesis and a change in epididymal growth. Previous reports documented the adverse effects of some effective drugs (e.g., sulfasalazine, testosterone, anabolic steroids, opioids, cyproterone acetic acid derivation, tramadol, sartan, and GnRH analogs) [27]. *E. brevicornum* Maxim (EbM) is a Chinese herb that possesses many pharmacological activities, including hormonal enhancement, immunological capacity regulation, antiosteoporosis, antitumor, antioxidation, antimaturity, and antiatherosclerosis. Moreover, over 2000 years, it has been used as an antirheumatic natural tonic and aphrodisiac in China, Japan, and Korea [9].

In this research, we present the risks of long-term use of TAM over 130 consecutive days. We tested its effects on male fertility by evaluating some andrological parameters on Wister male rats, namely, sperm motility, count, livability and abnormalities, serum testosterone, LH and FSH hormones, and serum estradiol (E2). These parameters are indicators that determine male ability to create feasible spermatozoa, some tissue-scavenging ability, and an apoptosis index (Bcl-2 and Bax gene expression). Additionally, we figured out the possible improvements in the aforementioned parameters using different doses *E. brevicornum* extract combined with TAM through the 65 days of treatment to detect their therapeutic effect.

The hypothalamus assumes a vital function in the maintenance of spermatogenesis by controlling FSH and LH secretion from the pituitary organ through GnRH [28]. At the same time, testosterone is integrated into the regulation of GnRH synthesis feedback and released through a long-circle input system [29]. Male maturity is dependent mainly on serum testosterone, LH and FSH concentrations, sperm count, and sperm quality. The regulated male sex hormone levels indicate the male reproductive brokenness. In the current study, Figure 4A reveals that blood testosterone levels delivered mainly in the testicles were substantially diminished (*p* ≤ 0.05) in the TAM-treated group compared to the control group. Testosterone is a significant androgen that plays a key role in sexual development, behavior, spermatogenesis, separation, and the maintenance of the adornment sex organs [30]. The synthesis and release of androgens are dependent on the pituitary gonadotropins, which are LH and FSH. Both LH and FSH are essential for testicular capacity and spermatogenesis. LH is the fundamental tropic controller of Leydig cells; without it, the androgen generation cannot be imagined [31].

Our findings show that the TAM-administered group had a marked decrease in serum concentrations of LH in the central pituitary organ (*p* ≤ 0.05). Serum FSH levels showed a substantial decrease in the TAM-administered group in contrast to the control group. Our findings of a reduction rather than an increase in circling LH in the TAM-treated group indicate that the drug was acting on the hypothalamus axis by suppressing LH release by the pituitary gland. Moreover, estradiol (E2), which has shown a controlled decrease in TAM-treated rats, is the overwhelming controller of FSH discharge in human males [32]. Estradiol is generated by the aromatase activity in the Leydig cells of the mammalian testis, as well as in certain germ and Sertoli cells of immature warm-blooded animals [33]. Raven et al. [34] suggested that the levels of peripheral E2 directly reflect the inhibitory tone used by gonadotropin-release estrogens and are significant determinants of testosterone and LH and FSH levels. E2 prevents the release of gonadotropin in men by acting on the pituitary and hypothalamus. However, the mean plasma E2 level expected to restore LH, FSH, and testosterone to the standard levels was not substantially the same as the mean E2 level. *E. brevicornum* extract at a dose of 2.43 g/kg/day for 130 successive days stimulated the most impressive values, in contrast with testosterone, LH, FSH, and E2 control rats, showing improvement in male fertility, which was confirmed by sperm motility, abnormalities, and count when related to control rats, where a marked rise in sperm count and motility could be seen, with a marked decrease in abnormalities. Groups 4 and 5 received TAM at 50 mg/kg/day for 65 days and TAM and *E. brevicornum* extract at doses of 0.81 g/kg/day and 2.43 g/kg/day for 65 days, respectively, resulting in remarkable improvements in the previously mentioned parameters compared to the TAM-treated group.

In folk medicine, *E. brevicornum* extract has been documented in traditional Chinese medicine for treating erectile dysfunction as it has testosterone mimetic activity and therapeutic potential in hypoandrogenism management [35]. Shindel et al. [13] reported the beneficial effect of *E. brevicornum* on erectile function after cavernous nerve damage. They revealed that rats treated with a low-dose of *E. brevicornum* resulted in a remarkable increase in the intracavernous pressure (ICP) to mean arterial pressure (MAP) ratio and area under the curve (AUC) to MAP ratio in comparison to control animals. They concluded that *E. brevicornum* might have neurotrophic effects on penile tissue in addition to its known phosphodiesterase type 5 (PDE-5)-inhibiting effects. Interestingly, high blood testosterone levels in high-dose *E. brevicornum*-treated rats show significant degradation when compared to control or low-dose *E. brevicornum*-treated rats. At the same time, no improvement in the serum LH has been documented in all treated groups.

Irregular morphology of spermatozoa was increased in TAM-treated groups, whereas this irregular morphology was improved in *E. brevicornum*-treated groups compared to control. The sperm characteristics shown in Figure 3 showed a significant reduction (*p* ≤ 0.05) in sperm count, motility, and percent of viability in TAM oral administration (50 mg/kg/day b.wt.) for 130 consecutive days in comparison to healthy animals. Our results are compatible with those reported by Nna and Osim [36], who indicated a significant increase in spermatozoa with abnormal morphology in all TAM (20 mg/kg/day b.wt.)-treated groups, with regard to the control animals. TAM was impressively capable of implementing its malignant activities in the production of the testis as well as the function and, thus, the regulation of serum hormonal levels that regulate the male richness [27,36,37]. Furthermore, Abdel-Hamid et al. [7] suggested that TAM may exhibit a beneficial effect on premature ejaculation therapy. Rats treated with *E. brevicornum* at a dose of 2.43 g/kg/day for 130 consecutive days elicited a remarkable increase in sperm motility and count, with a marked decrease in sperm abnormalities compared to the control or TAM-treated groups. Nantia et al. [38] reviewed the effect of *E. brevicornum* extract on mammalian fertility and documented that the herb extract improved sperm parameters, as well as the treatment of libido dysfunction, sexual asthenia, and erection. In addition, another study documented that the oral administration of lipid-based *E. brevicornum* extract improved the erectile function of aged rats [39].

Interestingly, this is the first study to demonstrate the efficacy of the *E. brevicornum*–TAM combination treatment on male infertility. Animals receiving TAM at 50 mg/kg/day for 65 days, followed by TAM and *E. brevicornum* at doses of 0.81 and 2.43 g/kg/day for 65 days, respectively, had a marked increase in sperm characteristics compared to TAM-treated animals (Figure 3), and an improvement was documented in the rodents receiving *E. brevicornum* at a dose of 2.43 g/kg/day. Tramadol administration at a dose of 50 mg/kg/day b.wt. for 130 consecutive days led to a substantial reduction in SOD activity and a remarkable rise in MDA levels when compared to the control group. MDA is the main reactive aldehyde that is known to have a toxic effect on cells [40]. All of these defensive antioxidant enzymes can work together and, therefore, protect against oxidative injury or damage caused by free radicals. These results are consistent with previous reports that show that TAM causes oxidative anxiety in testicular tissue [37,41,42]. In addition, TAM has a narcotic pain-relieving effect, which is usually recommended for moderate to severe pain at the usual dose of up to 200 mg/day, as indicated by Costa et al. [41]. TAM is also reported as one of the synthetic opioids that have significant cellular impacts through the expansion of lipid peroxidation that can be used as an indicator of cell injury caused by reactive oxygen species (ROS) [43]. However, the findings documented by Atici et al. [44] showed that morphine and TAM treatment afforded an elevated MDA level, suggesting an increased level of lipid peroxidation. In addition, reduced levels of glutathione (GSH) were observed in isolated rat hepatic cells incubated with different concentrations of opioids that resulted in cell necrosis and decreased GSH levels, as well as CAT, SOD, and glutathione peroxidase (GPx) activity. Recently, Nna and Osim [36] confirmed that SOD, CAT, GSH, and GPx were completely reduced, while MDA was increased mainly in all TAM (20 mg/kg/day b.wt.)-treated groups when compared with control.

Additionally, TAM administration at 50 mg/kg/day b.wt. for 130 consecutive days led to a remarkable rise in the NO levels in contrast with different groups. These data were supported by those obtained by Ahmed and Kurkar. [37], who revealed that TAM substantially enhanced nitric oxide testicular levels and lipid peroxidation when compared to the control group. Similarly, immunohistochemical studies demonstrated that TAM enhanced endothelial nitric oxide synthase (eNOS) expression in testicular tissues. *E. brevicornum* administration, at a dose of 2.43 g/kg/day for 130 successive days, resulted in a detectable increase in SOD activity and a marked decrease in both MDA and NO levels when compared to either control or TAM-treated groups. Zhao et al. [45] evaluated the in vitro antioxidant activity using a DPPH assay. The assay showed that phenolic compounds (e.g., p-hydroxybenzoic acid, gallic acid, vanillic acid, ferulic acid, rutin, caffeic acid, catechin, quercetin, and benzoic acid) isolated from EbM exhibit potent antioxidant activity. Moreover, they documented that *E. brevicornum* pretreatment for 24 h markedly enhanced cisplatin-actuated oxidative stress by diminishing the MDA and ROS levels, while increasing GSH levels in HEK-293 cells, as well as decreasing nuclear translocation and nuclear factor-kappa B (NF-κB) phosphorylation, followed by reduced iNOS, interleukin-1β (IL-1β), and tumor necrosis factor-α (TNF-α) secretions. It also decreased cellular apoptosis by reducing Bax and cleaved caspase-3/9 levels and enhancing the antiapoptotic protein Bcl-2 in the cells [46].

Nitric oxide (NO) is a reactive radical particle formed by oxidizing l-arginine guanidino nitrogen by NO synthase (NOS), and it is essential for the host defense strategy against several pathogens, such as parasites, fungi, viruses, and bacteria [47]. However, too much NO production can lead to the development of inflammatory disorders such as autoimmune disease and rheumatoid arthritis [48]. Although the exact mechanisms for controlling the anti-inflammatory activity of *E. brevicornum* have not yet been understood, Yuk et al. [49] showed that *E. brevicornum* water extract suppresses lipopolysaccharide-stimulated (LPS) proinflammatory mediator production, such as IL-10, IL-3, IL-12, IL-17, and NO in RAW264.7 rodent mononuclear phagocyte systems. These findings demonstrate that *E. brevicornum* possesses anti-inflammatory effects and may change macrophage-mediated inflammatory activation. Notably, Shindel et al. [13] reported that both Western blot and immunohistochemistry assays showed substantially higher positivity for nNOS expression and calponin in penile tissues of all *E. brevicornum*-treated animals. Histopathological assessment of testicular slices related to control animals revealed typical, unblemished seminiferous tubules with normal lining epithelium and stratified seminiferous epithelium with typical interstitial connective tissue. In comparison, TAM (50 mg/kg/day b.wt.)-treated rats demonstrated necrosis and seminiferous tubules degeneration, sloughing of the stratified seminiferous epithelium, spermatogenic cells, and Sertoli cells. There was exceptional necrosis of some spermatogenic cells, spermatogonia, spermatocytes, and spermatids that appeared with a pyknotic nucleus. Concerning the seminiferous tubules, focal disorder, irregularity, increased spaces between the tubules, and detached basement membrane of some tubules, as well as shrunken seminiferous tubules, were also detected.

Elnaga et al. [50] could not agree more as they noticed spermatogenic population depletion in most tubules, focal disorganization, anomaly, expanded spaces between the tubules, and detached basement membrane of some tubules, as well as shrunken seminiferous tubules. They documented that spermatogenic cells of animals that received 40 mg/kg/day TAM daily for 6 successive weeks were replaced by vacuoles, whereas other seminiferous tubules lack sperms and spermatids. Moreover, they detected darkly stained nuclei of spermatogonia and spermatocytes. They also observed vacuolated homogenous acidophilic material of the interstitium, Pyknotic nuclei, binucleated cells, vacuolated spermatogonia, and spermatocytes, as well as deeply stained nuclei of Leydig cells. Ghoneim et al. [51] revealed that TAM treatment for 4 weeks showed distinct histological changes. The seminiferous tubules existed in abnormal shape, broadly isolated from each other, and some tubules had ruptured without spermatozoa. Numerous vacuoles were observed between the multinucleated giant cells, apoptotic cells, and spermatogenic cells. Several inflammatory cells were shown in the intertubular tissues. In TAM (40, 80, 120, and 160 mg)-treated groups, severe testicular-diffused degradation, with multiple spermatocytes and spermatid giant cell formation, unaccompanied by spermatogenesis, was detected. Spermatocytes were mainly necrotic in lesser dosed samples with a recovery attempt, and inexhaustible cores have been shown by the regenerated cells. Micro spermatozoa and dystrophic calcification were found inside the lumen of seminiferous tubules. Histopathology injuries were affected by the overhaul dose of TAM before testicular tissue has been calcificated [52].

The current study examines the effects of chronic administration of TAM (50 mg/kg/day b.wt.) and *E. brevicornum* at a dose of 2.43 g/kg/day for 130 consecutive days and TAM at 50 mg/kg/day for 65 days, followed by concurrent usage of TAM and *E. brevicornum* at doses of 0.81 and 2.43 g/kg/day for 65 days, respectively. *E. brevicornum,* at a dose of 2.43 g/kg/day, illustrated normal testicular parenchyma, as shown in the control group. Tramadol treatment followed by concomitant use of TAM and *E. brevicornum,* at a dose of 0.81 g/kg/day, showed that testicular parenchyma appeared intact but with a slight pathological lesion within the epithelium of the seminar tubular epithelium. Furthermore, there were some destruction and sloughing of spermatogenic cells. Tramadol administration, followed by concurrent use of TAM and higher doses of *E. brevicornum,* showed that the testicular formation of parenchyma appeared to be homogeneous and healthy, apparently normal, with a typical arrangement of stratified seminiferous epithelium, and spermatogenic and Sertoli cells. Likewise, Leydig cells are claimed to be healthy and intact, without any pathological lesions.

## 5. Conclusions

In conclusion, the present investigation reveals the significant potential role of *E. brevicornum* extract in alleviating the negative impacts of tramadol in male Wister rats by regulating antioxidant functioning and hormonal and apoptotic markers. *E. brevicornum* extract application prevented oxidative damage to tramadol by upregulating the NO level and antioxidant-functioning, with a subsequent decline in the oxidant status. As a result, *E. brevicornum* extract may cause a protective action and enhancement of testicular tissue function, increasing sperm count and motility in the rat model of male infertility. The present study suggests that the supplementation of *E. brevicornum* extract may improve spermatogenesis by reducing oxidative stress in the luteinizing hormone-releasing hormone agonist-induced rat model of male infertility. Therefore, *E. brevicornum* extract application can be exploited to improve anti-infertility agents by its active involvement in key regulatory functions.

## Figures and Tables

**Table 1:** Oligonucleotide sequences for interest and reference genes.

Gene	Forward Primer (5′–3′)	Reverse Primer (5′–3′)	Accession No	Product Size	Ref.
Bax	CGAATTGGCGATGAACTGGA	CAAACATGTCAGCTGCCACAC	NM_017059.2	109	[25]
Bcl2	GACTGAGTACCTGAACCGGCATC	CTGAGCAGCGTCTTCAGAGACA	NM_016993.1	135	[25]
GAPDH	GGCACAGTCAAGGCTGAGAATG	ATGGTGGTGAAGACGCCAGTA	NM_017008.4	143	[25]
